# Risk of cervical lesions in high-risk HPV positive women with normal cytology: a retrospective single-center study in China

**DOI:** 10.1186/s13027-020-00291-x

**Published:** 2020-05-21

**Authors:** Zhiling Wang, Ting Liu, Yunjian Wang, Ying Gu, Hui Wang, Jingkang Liu, Baoxia Cui, Xingsheng Yang

**Affiliations:** 1 Department of Gynecology, Qilu Hospital of Shandong University, Jinan, China; 2 West China School of Medicine, Sichuan University, Chengdu, China

**Keywords:** Uterine cervical neoplasms, Human papillomavirus, Cytology, Viral load

## Abstract

**Background:**

To investigate high-risk HPV (hr-HPV) genotype distributions and the association between hr-HPV infection with severity of the cervical lesions in women with normal cytology.

**Methods:**

In this cross-sectional study, the result of the hr-HPV test and biopsy of colposcopy of women with normal cytology from January 2012 to January 2019 were analyzed. The detection rate of high-grade squamous intraepithelial lesion (HSIL) and cervical cancer were calculated among different hr-HPV genotypes, viral load group, and age groups.

**Results:**

Five thousand eight hundred eighty women were enrolled in this study. Overall, 59.97% had normal histological results, 19.32% had HSIL, and 1.07% had cervical cancer. The detection rate of HSIL or worse (HSIL+) in women with single HPV16(34.00%), HPV31(27.50%), HPV33(25.58%), and HPV52(20.88%) infection were higher significantly than single HPV18 (15.59%) infection, respectively. The HSIL+ detection rate between HPV16 single infection and multiple infections (excluding HPV18) was no significant difference (34% vs 35.47%, *P* = 0.638), contrary to HPV18(12.59% vs 21.67%, *P* = 0.022). In women without HPV16/18 infections, HSIL+ detection rates for single, double, and triple or more hr-HPV infections were 12.28, 20.31, and 37.50%, the risk of detection of HSIL+ significantly increasing. With the hr-HPV DNA load increases, the risk of detection of HSIL+ (χ2 = 91.01, *P* < 0.0001) and invasive cervical cancer (χ2 = 5.757, *P* = 0.016) increase. In age < 30, 31–40, 41–50, 51–60, > 60 group, HSIL+ detection rate were 24.80%、22.10%、19.59%、14.29, and 12.61%, respectively.

**Conclusion:**

Women who have normal cytology with HPV 16/18/31/33/52/58 infections, multiple HPV infections and high viral load, have a higher detection rate of HSIL+.

## Introduction

High-risk HPV (hr-HPV) infection is the main etiological factor for the development of cervical neoplasia [1], and routine cervical cancer screening includes hr-HPV and cervical cytology tests. Previous randomized controlled trials [2–6] have shown that the strengths of HPV testing are higher sensitivity and negative predictive value than cervical cytology. Given that cumulative incidence of cervical intraepithelial neoplasia (CIN) 3 or cancer 5 years after an HPV-negative test was lower than the risk 3 years after a negative Pap test [6, 7], HPV testing was introduced as the supplementary test in conventional cytology-based screening, even gradually replacing cytology-based screening in primary screening for cervical cancer in some countries [8, 9]. As HPV testing is widely used, the optimal clinical management of women with HPV infection, especially combined normal cytology, remains a challenge. The guidelines recommend managing HPV-positive/cytology-negative results by return testing at 1 year or HPV genotyping for HPV16 and HPV18 [10]. If women have negative cytology but HPV16 and HPV18 infection(s), immediate colposcopy is recommended instead of a 1-year return. HPV16 has higher relevance with cervical lesions than other hr-HPV types [9, 11, 12]. But the reason for choosing HPV18 is uncertain, partly because HPV18 infection has a correlation with cervical adenocarcinoma, which cannot be well detected by cytology [11]. The correlation with other types HPV is not clear yet. What’s more, whether viral load can be used as a marker to predict the severity of cervical lesions is currently controversial [13].

Therefore, this study was designed to retrospectively analyze data for up to 7 years, to find the correlation between the severity of cervical lesions and hr-HPV genotypes, viral loads, and ages in HPV-positive/cytology-negative females in China.

## Materials and methods

### Study population

This study was cross-sectional investigation based on data from women who accepted Thinprep cytologic test (TCT) test, HPV test, and colposcopy at Qilu Hospital of Shandong University from January 2012 to January 2019, after approval of the Qilu Hospital of Shandong University【2018(054)】. Clinical information of women who met the inclusion criteria was collected retrospectively.

Inclusion criteria: (1) women with normal cytology and hr-HPV infection(s); (2) women accepted colposcopic examination and underwent cervical biopsy under colposcopic guidance.

Exclusion criteria: (1) women with history of treatments to cervical lesions, such as cervical surgery and medicine, et al.; (2) women with incomplete cervical cervix; (3) women with malignant tumors; (4) women with autoimmune diseases or receiving immunotherapy; (5) women with pregnancy; (6) women who had received HPV vaccine.

### Cytology test

Specimen collection, specimen preparations, and results of the tests are performed, according to the instructions of the manufacturers, respectively.

One of those HPV tests could be chosen:

1. Hybrid Capture 2(HC2) High-Risk HPV DNA Test (Qiagen, Gaithersburg, MD, USA): semi-quantitative detection of 13 h-HPV types: 16, 18, 31, 33, 35, 39, 45, 51, 52, 56, 58, 59 and 68. Samples were considered as positive if the ratio relative-light-unit (RLU)/cut-off(CO) was > 1.0 (equivalent to 1.0 pg HPV DNA/mL or 100,000 HPV copies/mL) as described in package insert.
2. the Cobas 4800 System (Roche Diagnostics Corporation, Indianapolis, Indiana): Qualitative detection of HPV 16, HPV 18 and 12 h-HPV (a pool of 12 other hr-HPV types: including HPV 31,33,35,39,45,51,52,56,58,59,66 and 68).
3. the HPV GenoArray test (Hybribio Biotechnology Ltd. Corp, Chaozhou, Guangdong Province, China): qualitative detection of 15 kinds of hr-HPV types: 16, 18, 31, 33, 35, 39, 45, 51, 52, 53, 56, 58, 59, 66 and 68; 6 kinds of low risk HPV (lr- HPV) types: 6, 11, 42, 43, 44 and 81(CP8304).

### HPV test

Specimen collection, specimen preparations, and results of the tests are performed, according to the instructions of the manufacturers, respectively. ThinPrep (Hologic Inc., Marlborough, Massachusetts, USA)preparations was used, and the cytological diagnosis was performed by experienced cytopathologists, according to the Bethesda system TBS 2001.

### Colposcopy and guided cervical biopsies

All women, enrolled in this study, underwent colposcopy and guided cervical biopsies according to the American Society for Colposcopy and Cervical Pathology Colposcopy Standards recommendations [14]. The histologic diagnosis was regarded as gold standards for this study and was based on the consensus diagnosis of two experienced gynecologic pathologists who were blinded to results of TCT, HPV test, and colposcopic examination. The results of biopsy reports include: normal; low-grade squamous intraepithelial lesion (LSIL) (including CIN 1), high-grade squamous intraepithelial lesion (HSIL) (including CIN 2–3), and cervical cancer. HSIL or worse (HSIL+) includes HSIL and cervical cancer.

### Statistical analysis

Statistical analyses were performed using the SAS 9.4 (SAS Institute, Cary, NC) for windows. The categorical variables were expressed by a percentage (%). The binary Logistic regression model was used to analyze the detection rate of HSIL+ and cervical cancer in different hr-HPV genotype groups. The relationships between the HPV viral load and the detection rate of HSIL+ were analyzed by the Cochran-Armitage Trend test. Results were considered statistically significant at *p*-values < 0.05.

## Result

### Characteristics of the study population

**Fig. 1:**
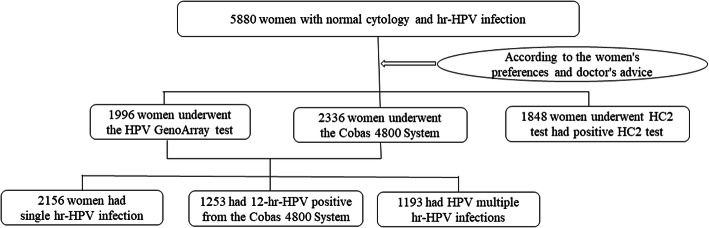
Flow chart of women with normal cytology choosing hr-HPV test. HPV= human papillomavirus. hr-HPV= high-risk human papillomavirus. HC2= Hybrid Capture 2

A total of 5880 women with normal cytology and hr-HPV infection accepted colposcopic biopsy were enrolled in this study. 4332 women underwent HPV genotyping test and 1548 women were hr-HPV DNA positive by HC2 hr-HPV DNA test (Fig. 1). The median age was 39 years (26–81), and the average age was 39.29 ± 9.72 years. They were divided into 5 age groups.

**Fig. 2:**
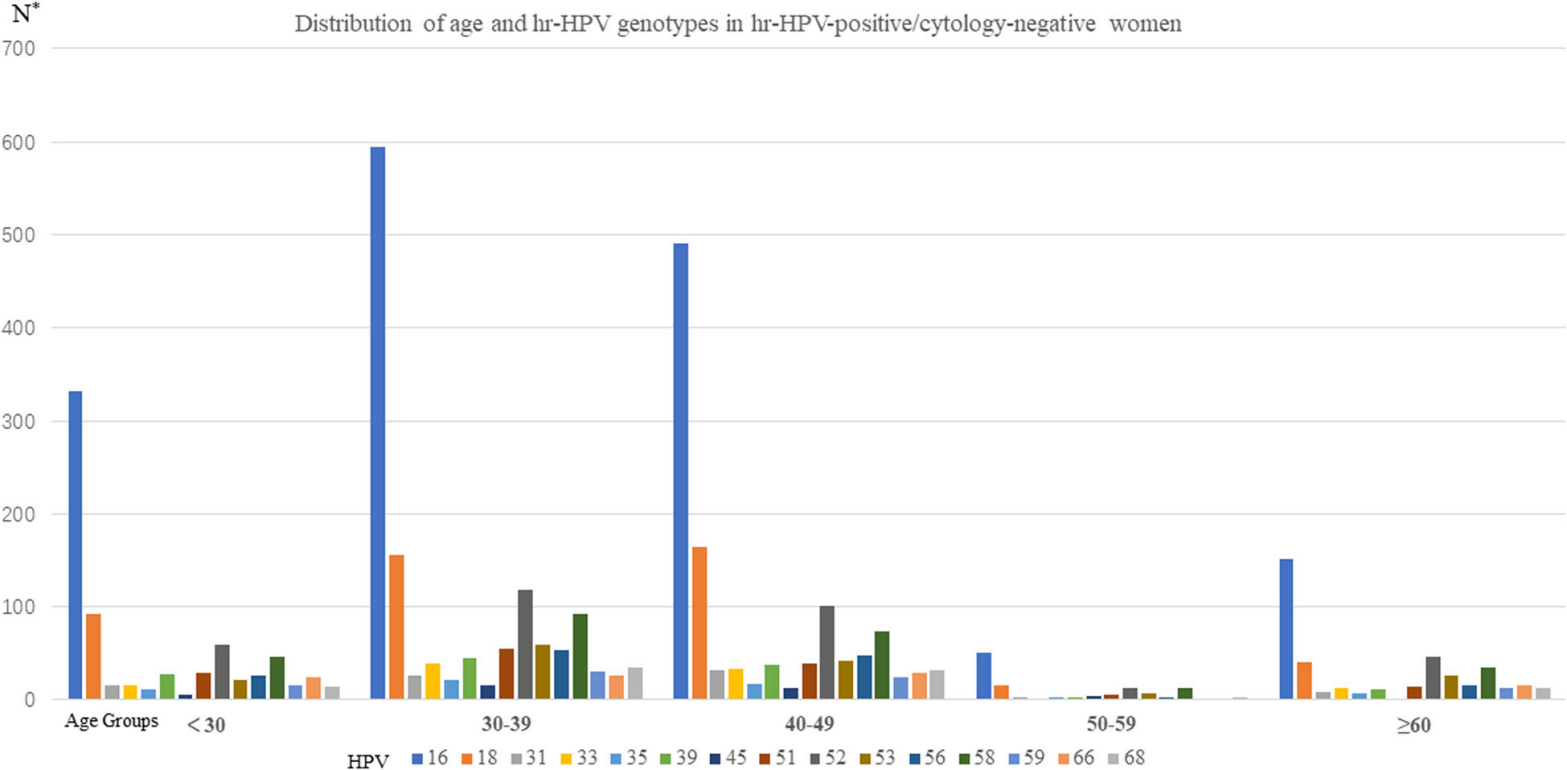
Distribution of age and hr-HPV genotypes in hr-HPV-positive/cytology-negative women. HPV= human papillomavirus. hr-HPV=high-risk human papillomavirus. *= The sum of women with the type-specific hr-HPV infection(s) and it would be counted multiple times if the person has multiple hr-HPV infections

The prevalence of HPV infection among all age groups showed in Table 1 and Fig. 2. HPV16 and HPV18 were the most common, followed by HPV52 and 58, in all age groups. Among women with normal cytology and HPV infection, 3526(59.97%) women had normal pathology, 1155(19.64%) women had LSIL, 1136(19.32%) had HSIL. The remaining 63(1.07%) woman was diagnosed with cervical cancer, including 17 women with positive HC2-hr-HPV-DNA-test results, 31 women with single HPV16 infection, 4 women with HPV 18 infection, 8 women with multiple HPV 16 infections without HPV18, 1 woman with HPV 16 and HPV18 infections, and 2 women with HPV58 infection.

**Table 1:** Distribution of age and hr-HPV genotypes in hr-HPV-positive/cytology-negative women

Age	hr-HPV genotypes																	N
	16	18	31	33	35	39	45	51	52	53	56	58	59	66	68	12-hr^a^	HC2^b^	
**<30**	332	92	16	15	11	27	5	29	59	21	26	46	15	24	14	286	223	992
**30–39**	595	156	26	38	22	45	15	54	118	59	53	92	30	25	34	578	575	2140
**40–49**	490	165	31	33	17	37	13	39	101	41	47	73	24	29	32	509	496	1868
**50–59**	50	16	2	1	3	3	4	5	13	7	3	12	1	1	3	58	47	182
**≥60**	152	40	8	13	7	11	1	14	46	25	15	35	12	16	12	195	207	698
**N**^c^	1619	469	83	100	60	123	38	141	337	153	144	258	82	95	95	1626	1548	5880

*HPV* human papillomavirus

*hr-HPV* high-risk human papillomavirus

^a^=12 hr-HPV (a pool of 12 other hr-HPV types: including HPV 31,33,35,39,45,51,52,56,58,59,66 and 68) from the Cobas 4800 System

^b^=13 hr-HPV (16, 18, 31, 33, 35, 39, 45, 51, 52, 56, 58, 59 and 68) by Hybrid Capture 2(HC2)High-Risk HPV DNA Test

^c^= The sum of women with the type-specific hr-HPV infection(s) and it would be counted multiple times if the person has multiple hr-HPV infections

### Single hr-HPV infection and histopathology in hr-HPV-positive/cytology-negative women

The detection rates of HSIL+ are different among different hr-HPV types. The rate of HPV16 is highest(34.00%), followed by HPV31(27.50%), HPV33(25.58%), HPV52(20.77%), HPV35(17.39%), HPV58(16.18%), HPV18(12.59%), HPV51(6.25%), HPV66(4.88%), HPV45(4.76%), HPV39(4.62%), HPV56(4.69%), HPV68(4.17%), HPV59(3.03%), HPV53(1.28%). Compared with single HPV18 infection, the detection rate of HSIL+ was higher in women with single HPV16/31/33/52 infection, with OR values of 3.576(95% CI:2.442–5.238), 2.633(95% CI:1.205–5.753), 2.386(95% CI:1.101–5.172), 1.819(95% CI:1.096–3.02), respectively. There was no significant difference in the detection rates of HSIL+ between single HPV35/39/45/51/56/58/59/66/68 and single HPV18 infection. The detection rate of HSIL+ with single HPV53 infection was lower than that in HPV18, while OR value was 0.090 (95% CI: 0.012–0.670). (Table 2).

**Table 2:** Distribution of hr-HPV genotype and histological results among single-hr-HPV-positive/cytology-negative women

HPV Types	Histological Results						
	TOTAL	NORMAL(n,%)	LSIL(n,%)	HSIL(n,%)	CA(n,%)	HSIL+(n,%)	HSIL+OR(95% CI)
**16**	1047	522 (49.86)	169 (16.14)	325 (31.04)	31 (2.96)	356 (34.00)	3.576 (2.442–5.238)
**18**	270	175 (64.81)	61 (22.59)	30 (11.11)	4 (1.48)	34 (12.59)	1
**31**	40	19 (47.50)	10 (25.00)	11 (27.50)	0 (0.00)	11 (27.50)	2.633 (1.205–5.753)
**33**	43	24 (55.81)	8 (18.60)	11 (25.58)	0 (0.00)	11 (25.58)	2.386 (1.101–5.172)
**35**	23	14 (60.87)	5 (21.74)	4 (17.39)	0 (0.00)	4 (17.39)	1.461 (0.469–4.554)
**39**	65	49 (75.38)	13 (20.00)	3 (4.62)	0 (0.00)	3 (4.62)	0.336 (0.100–1.130)
**45**	21	16 (76.19)	4 (19.05)	1 (4.76)	0 (0.00)	1 (4.76)	0.347 (0.045–2.670)
**51**	64	44 (68.75)	16 (25.00)	4 (6.25)	0 (0.00)	4 (6.25)	0.463 (0.158–1.355)
**52**	183	115 (62.84)	30 (16.39)	38 (20.77)	0 (0.00)	38 (20.77)	1.819 (1.096–3.020)
**53**	78	62 (79.49)	15 (19.23)	1 (1.28)	0 (0.00)	1 (1.28)	0.090 (0.012–0.670)
**56**	64	41 (64.06)	20 (31.25)	3 (4.69)	0 (0.00)	3 (4.69)	0.341 (0.101–1.149)
**58**	136	86 (63.24)	28 (20.59)	20 (14.71)	2 (1.47)	22 (16.18)	1.340 (0.749–2.395)
**59**	33	22 (66.67)	10 (30.30)	1 (3.03)	0 (0.00)	1 (3.03)	0.217 (0.029–1.639)
**66**	41	29 (70.73)	10 (24.39)	2 (4.88)	0 (0.00)	2 (4.88)	0.356 (0.082–1.542)
**68**	48	37 (77.08)	9 (18.75)	2 (4.17)	0 (0.00)	2 (4.17)	0.302 (0.070–1.300)
**N**^a^	2156	1255 (58.21)	408 (18.920	456 (21.15)	37 (1.72)	493 (22.87)	–

*HPV* human papillomavirus

*hr-HPV* high-risk human papillomavirus

*LSIL* low-grade squamous intraepithelial lesion

*HSIL* high-grade squamous intraepithelial lesion

*CA* cervical cancer

*HSIL+* high-grade squamous intraepithelial lesion or worse

*OR* odds ratio

*CI* confidence interval

^a^=The total number of women with hr-HPV positive in the corresponding histological results groups

### Multiple hr-HPV infections and histopathology in hr-HPV-positive/cytology-negative women

There was no significant difference in the detection rate of HSIL+ between single infection of HPV16 and multiple infections with HPV16 (excluding HPV18) (*P* = 0.599), not same as HPV18 (*P* = 0.022).

In hr-HPV-positive/cytology-negative women without HPV16/18 infection(s), the HSIL+ detection rates in single hr-HPV infection, double infections and triple or more infections were 12.28, 20.31 and 37.50%, respectively. Compared with the detection rate of HSIL+ in single hr-HPV infection (excluding HPV16/18), OR of the detection rate of HSIL+ in double and triple or more HPV infection was 1.821(95% CI:1.212–2.738) and 4.287(95% CI:2.188–8.400), respectively. Moreover, there was a significant difference between double and triple or more HPV infections (excluding HPV16/18) (*P* = 0.019). (Table 3).

**Table 3:** Distribution of hr-HPV genotypes and histological results among hr-HPV-positive/cytology-negative women

HPV Type(s)	Histological Results						
	TOTAL	NORMAL(n,%)	LSIL(n,%)	HSIL(n,%)	CA(n,%)	HSIL+(n,%)	HSIL+ OR(95% CI)
**HPV infection(s) with 16 and/or 18**							
HPV16	1047	522 (49.86)	169 (16.14)	325 (31.04)	31 (2.96)	356 (34.00)	
HPV18	270	175 (64.81)	61 (22.59)	30 (11.11)	4 (1.48)	34 (12.59)	
16 + other(s)^a^	492	213 (43.29)	105 (21.34)	166 (33.74)	8 (1.63)	174 (35.37)	
18 + other(s)^a^	120	56 (46.67)	38 (31.67)	26 (21.67)	0 (0.00)	26 (21.67)	
16 + 18 + other(s) ^a^	79	30 (37.97)	17 (21.52)	31 (39.24)	1 (1.27)	32 (40.51)	
**No. of hr HPV type(s) without HPV16 or 18**							
1 type^b^	839	558 (66.51)	178 (21.22)	101 (12.04)	2 (0.24)	103 (12.28)	1
2 types^c^	192	118 (61.46)	35 (18.23)	39 (20.31)	0 (0.00)	39 (20.31)	1.821 (1.212–2.738)
≥ 3 types^d^	40	19 (47.50)	6 (15.00)	15 (37.50)	0 (0.00)	15 (37.50)	4.287 (2.188–8.400)
**Unknown type(s)**							
HC2^e^	1548	984 (63.57)	300 (19.38)	247 (15.96)	17 (1.10)	264 (17.05)	
12 hr-HPV^f^	1253	851 (67.92)	246 (19.63)	156 (12.45)	0 (0.00)	156 (12.45)	
**Total**							
N^g^	5880	3526 (59.97)	1155 (19.64)	1136 (19.32)	63 (1.07)	1199 (20.39)	

*HPV* human papillomavirus

*hr-HPV* high-risk human papillomavirus

*LSIL* low-grade squamous intraepithelial lesion

*HSIL* high-grade squamous intraepithelial lesion

*CA* cervical cancer

*HSIL+* high-grade squamous intraepithelial lesion or worse

*OR* odds ratio

*CI* confidence interval

^a^=hr-HPV genotypes except HPV16 or 18

^b^= single hr-HPV infection without HPV16 or 18

^c^= double hr-HPV infections HPV16 or 18

^d^= triple or more hr-HPV infections HPV16 or 18

^e^= infection(s) with a pool of 12 other hr-HPV types (including HPV 31,33,35,39,45,51,52,56,58,59,66 and 68) that did not know the specific type of HPV (from Cobas 4800 HPV test)

^f^=13 hr-HPV types (16, 18, 31, 33, 35, 39, 45, 51, 52, 56, 58, 59 and 68) tested by Hybrid Capture 2(HC2)High-Risk HPV DNA Test

^g^=The total number of people with hr-HPV positive in the corresponding histological results groups

### Viral load and histopathology in hr-HPV-positive specimen

One thousand five hundred forty-eight women were hr-HPV DNA positive by HC2-hr-HPV DNA test, and they were divided into 3 groups by viral load: low viral load group (1 ≤ RLU/CO < 10, *n* = 516), medium viral load group (10 ≤ RLU/CO < 100, *n* = 450), and high viral load group (RLU/CO ≥100, *n* = 582). The HSIL+ detection rates were 5.43, 17.33 and 27.15% in 3 groups, respectively; with the increase of viral load, the higher the risk was(χ2 = 91.01, P<0.0001). The detection rates of invasive cancer in 3 groups were 0.19, 1.33 and 1.72%, respectively. As the same, viral load is proportional to the risk(χ2 = 5.757, *P* = 0.016).(Table 4).

**Table 4:** Histological results and viral load among hr-HPV-positive/cytology-negative women

Viral Load	Histological Results					
	TOTAL	NORMAL (n,%)	LSIL (n,%)	HSIL (n,%)	CA (n,%)	HSIL+ (n,%)
1 ≤ RLU/CO<10	516	409 (79.26)	79 (15.31)	27 (5.23)	1 (0.19)	28 (5.43)
10 ≤ RLU/CO<100	450	281 (62.44)	91 (20.22)	72 (16.00)	6 (1.33)	78 (17.33)
RLU/CO ≥100	582	294 (50.52)	130 (22.34)	148 (25.43)	10 (1.72)	158 (27.15)
N^a^	1548	984 (63.57)	300 (19.38)	247 (15.96)	17 (1.10)	264 (17.05)

*HPV* human papillomavirus

*hr-HPV* high-risk human papillomavirus

*RLU/CO* the ratio relative-light-units /cut-off

*LSIL* low-grade squamous intraepithelial lesion

*HSIL* high-grade squamous intraepithelial lesion

*CA* cervical cancer

*HSIL+* high-grade squamous intraepithelial lesion and cervical cancer

^a^=The total number of people with different RLU in the corresponding histological results groups

### Age and histopathology in hr-HPV-positive/cytology-negative women

**Fig. 3:**
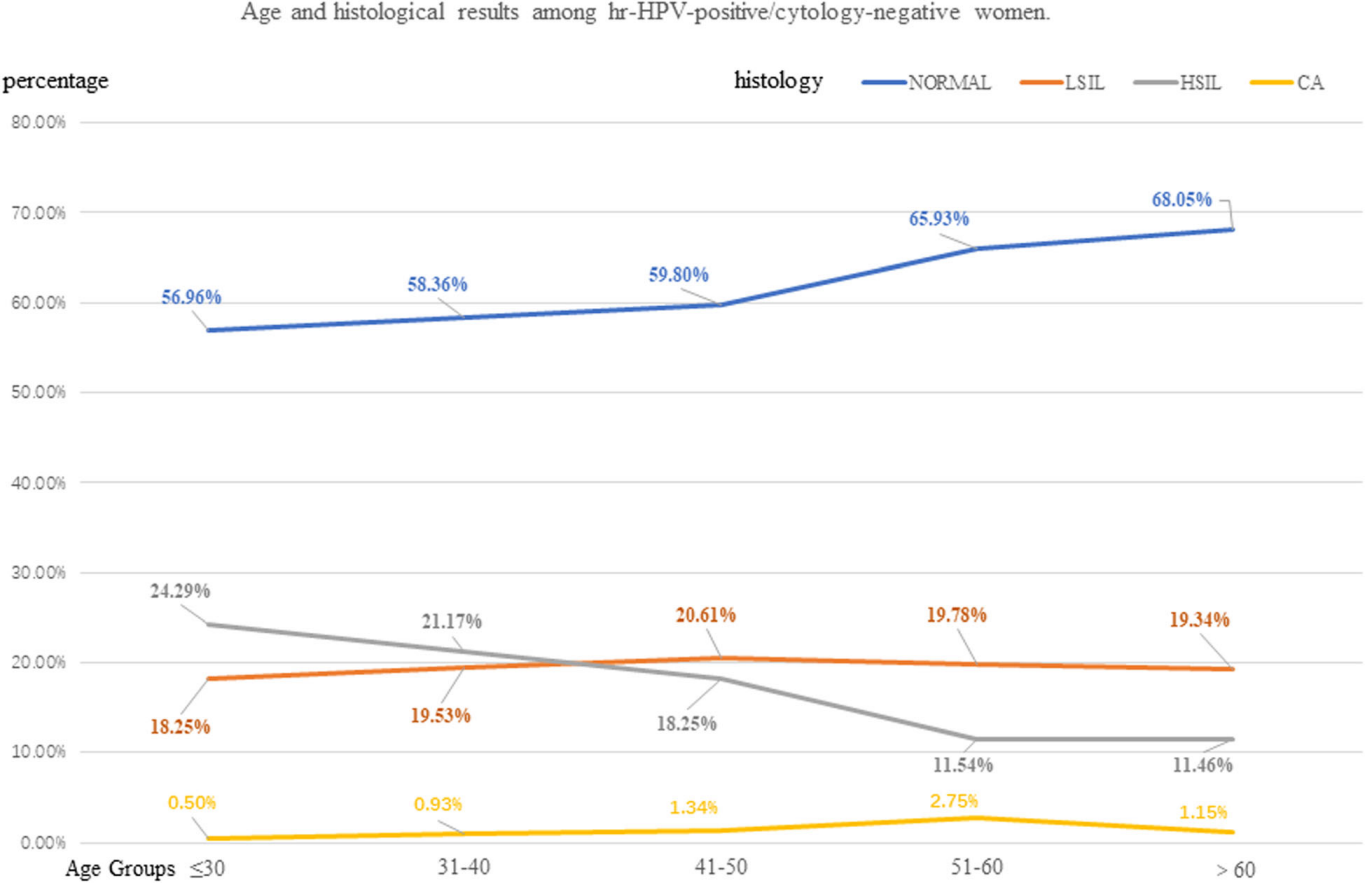
Age and histological results among hr-HPV-positive/cytology-negative women. HPV=human papillomavirus. hr-HPV= high-risk papillomavirus

HSIL+ detection rate changed by different age groups. In age < 30, 31–40, 41–50, 51–60, > 60 group, HSIL+ detection rate were 24.80%、22.10%、19.59%、14.29, and 12.61%, respectively. The cancer detection rate was the highest in the age 54–60 group, which was 2.75%. However, the detection rate of cancer in age < 30 group (0.50%) was lowest (Table 5, Fig.3).

**Table 5:** Age and histological results among hr-HPV-positive/cytology-negative women

AGE	Histological Results					
	TOTAL	NORMAL(n,%)	LSIL(n,%)	HSIL(n,%)	CA(n,%)	HSIL+(n,%)
**<30**	992	565 (56.96)	181 (18.25)	241 (24.29)	5 (0.50)	246 (24.80)
**31–40**	2140	1249 (58.36)	418 (19.53)	453 (21.17)	20 (0.93)	473 (22.10)
**41–50**	1868	1117 (59.80)	385 (20.61)	341 (18.25)	25 (1.34)	336 (19.59)
**51–60**	182	120 (65.93)	36 (19.78)	21 (11.54)	5 (2.75)	26 (14.29)
**>60**	698	475 (68.05)	135 (19.34)	80 (11.46)	8 (1.15)	88 (12.61)
**N**^**a**^	5880	3526 (59.97)	1155 (19.64)	1136 (19.32)	63 (1.07)	1199 (20.39)

*HPV* human papillomavirus

*hr-HPV* high-risk human papillomavirus

*LSIL* low-grade squamous intraepithelial lesion

*HSIL* high-grade squamous intraepithelial lesion

*CA* cervical cancer

*HSIL+* high-grade squamous intraepithelial lesion and cervical cancer

^a^=The total number of people with different ages in the corresponding histological results groups

## Discussion

Tests designed to stratify risks, such as HPV tests and cytological tests, play a vital role in managing patients. Most of the HPV-negative women is associated with a very low risk for developing cervical cancer [4–7]. One of the clinicians’ work is to identify women at high risk of cervical lesions from HPV-positive and cytology-negative women.

This study chose HSIL+ as the primary endpoint, because the purpose of cervical cancer screening is to detect easily treatable precancerous lesions, most commonly defined as CIN2 and greater. And choosing CIN3 + as the endpoint would miss some invasive cancers which developed from a part of CIN2, although it will increase the rate of referral of colposcopy. However, the primary endpoint of HSIL + is not ideal enough. One of the reasons is that CIN2 is an equivocal diagnosis of precancer, part of CIN2 can regress spontaneously, and the rest may develop to CIN3 or even invasive cancer [15].

The guidelines recommend that women with normal cytology should undergo a colposcopy examination immediately if they have HPV16/18 infection, rather than return testing at 1 year [10]. However, a 5-year study showed that non-HPV 16/18-positive women with the normal cytological result also had an 11.4% risk of HSIL [16]. Another study also found that some of the 12 non-HPV16/18 genotypes had the same risk for developing to HSIL as HPV16/18 [17]. And this study investigated the difference in the detection rate of HSIL+ in different genotypes in women with normal cytology by Binary Logistic Regression Model, based on the HSIL+ detection rate of HPV18.

HPV16 is the most common carcinogenic genotype, even 2–3 times more than other HPV genotypes [11, 12]. This study found that the risk of HSIL+ in HPV16 positive women was higher than that of HPV18(OR:3.576, 95% CI:2.442–5.238), and the high carcinogenicity of HPV16 was verified again. HPV31, HPV33, and HPV52 showed a higher detection rate of HSIL+ than HPV18. A 12-year follow-up study in Denmark showed that the absolute risk of CIN3+ in patients with HPV31 positive was 14.3% (CI: 9.1–19.4) [18]. Previous studies showed HPV33 was a relative of HPV16 [19], and HPV33 infection was associated with high absolute risks for progression to high-grade cervical lesions [20–22].

This study found that HPV 52 and HPV58 were the most common type of HPV infection apart from HPV16/18, which was same with previous studies showed HPV 52 and HPV58 were common in Asia, including Korea [23–25] and China [26–28]. Recently, high carcinogenicity of HPV58 and HPV45 has attracted much clinicians’ attention. A meta-analysis showed that the pattern for HPV58 in Eastern Asia was different than elsewhere, and HPV58 continues to rise in the proportion of cervical diseases at all levels, accounting for 10.2% ± 3.9% of all invasive cervical cancer with HPV infection [29]. Another study showed that HPV-58 is more prominent in HSIL and invasive cervical cancer patients in Japan [30]. In this study, in women with single hr-HPV infection, excluding HPV16/18, cervical cancer only occurred in 2 women infected with HPV58, consistent with previous studies in southwestern China that HPV58 is common in ICC [31]. HPV45 is a close relative of HPV18 [19, 32], and some studies reported that HPV45 was the third most common genotype involved in invasive cervical carcinoma, found in 6% of cervical cancers [33]. However, this study did not observe the high carcinogenicity of HPV45 in women with hr-HPV infection and normal cytology, which was related to the abnormality of cytology in women infected with HPV45. And a study reported that only 0.5% of women with HPV45 infection had normal cytology [34].

Multivariate analysis showed that the risk of cervical cancer was 19.9 times higher in women with single HPV infection, and 31.8 times higher in those with multiple HPV infection [35]. This suggests that multiple infections have a cumulative effect on developing to HSIL+. This study analyzed the association between the detection rate of HSIL+ and multiple hr-HPV infections, excluding HPV16/18. Compared with single hr-HPV infection, the detection rate of HSIL+ in double hr-HPV infections (OR: 1.821, 95% CI:1.212–2.738) and ≥ triple hr-HPV infections (OR:4.287, 95% CI:2.188–8.400) increased significantly, and there was a significant difference in the detection rate of HSIL + between double hr-HPV infection and ≥ triple hr-HPV infection (*P* = 0.019), which means that more hr-HPV genotypes infected in women, the higher the HSIL detection rate.

It is controversial whether the viral hr-DNA load can be used as a reliable marker to predict cervical lesions. Previous studies have reported that HPV viral load has been associated with persistence of HPV infection and subsequent cervical precancerous lesions [13, 36, 37]. However, a research showed that the high viral load of 13 h-HPV types could not predict HSIL+ [38]. There were little previous researches to analyze the correlation between the severity of cervical lesions and viral DNA load in women with normal smears. A study reported that the level of viral load not predictive of HPV persistence or the development of cervical intraepithelial neoplasia and only HPV16 infection was significantly more likely to persist and to develop CIN in women under 30 with normal cytology [39, 40]. This study found that the higher viral load, the higher the detection rate of HSIL+ in women with normal cytology(χ2 = 91.01, *P*<0.0001), same as the detection rate of cervical cancer (χ2 = 5.757, *P* = 0.016), which may be correlated to the fact that patients with low-load HPV infection are more likely to be eliminated, while those with high-load HPV infection are more difficult to eliminate [41].

This study found that the absolute value of HSIL+ detection rate decreased with age, while the absolute value of cancer detection rate increased with age, in women with normal cytology and hr-HPV infection. The time of hr-HPV infection is short, and cervical cell morphology has not changed in young women, what’s more, the proportion of CIN2 that can regress is higher in young women [14]. However, hr-HPV infection time is longer, and the proportion of CIN2 that progresses to CIN3 or invasive cancer is higher, while the percentage of CIN2 that can regress is smaller, in older women [14, 40]. Therefore, older women had lower HSIL+ detection rate and higher cervical cancer detection rate in women with normal cytology and HPV infection.

## Conclusion

In conclusion, about one in five hr-HPV-positive/cytology-negative women are diagnosed with HSIL+. Women with HPV 16/18/31/33/52/58 infections, multiple HPV infections whether combined with HPV16/18 infections, as well as high viral load, have a higher detection rate of HSIL+, although they have normal cytology.

## Limitation

This study was a cross-sectional study without follow-up and it could not estimate the cumulative risk of cervical lesions within a period. The population enrolled in this study is the patients who underwent colposcopy in Qilu Hospital, Shandong University. So, the results of this study can not be directly applied to the general screening population, but it has certain reference significance for the diagnosis and treatment of patients coming to the hospital. Besides, we can not ignore the heterogeneity of 3 HPV assays and specifically analyze women infected with 12 h-HPV.

## Data Availability

We declared that materials described in the manuscript, including all relevant raw data, will be freely available to any scientist wishing to use them for non-commercial purposes, without breaching participant confidentiality. You can contact the corresponding author through the email to get the data.

## References

[1] Walboomers JM, Jacobs MV, Manos MM, Bosch FX, Kummer JA, Shah KV (1999). Human papillomavirus is a necessary cause of invasive cervical cancer worldwide. J Pathol.

[2] Bulkmans NW, Berkhof J, Rozendaal L, van Kemenade FJ, Boeke AJ, Bulk S (2007). Human papillomavirus DNA testing for the detection of cervical intraepithelial neoplasia grade 3 and cancer: 5-year follow-up of a randomised controlled implementation trial. Lancet.

[3] Sankaranarayanan R, Nene BM, Shastri SS, Jayant K, Muwonge R, Budukh AM (2009). HPV screening for cervical cancer in rural India. N Engl J Med.

[4] Ronco G, Giorgi-Rossi P, Carozzi F, Confortini M, Dalla Palma P, Del Mistro A (2010). New Technologies for Cervical Cancer Screening (NTCC) working group. Efficacy of human papillomavirus testing for the detection of invasive cervical cancers and cervical intraepithelial neoplasia: a randomised controlled trial. Lancet Oncol.

[5] Ogilvie GS, van Niekerk D, Krajden M, Smith LW, Cook D, Gondara L (2018). Effect of screening with primary cervical HPV testing vs cytology testing on high- grade cervical intraepithelial neoplasia at 48 months: the HPV FOCAL randomized clinical trial. JAMA.

[6] Ronco G, Dillner J, Elfström KM, Tunesi S, Snijders PJ, Arbyn M (2014). Efficacy of HPV-based screening for prevention of invasive cervical cancer: follow-up of four European randomised controlled trials. Lancet.

[7] Dillner J, Rebolj M, Birembaut P, Petry KU, Szarewski A, Munk C (2008). Long-term predictive values of cytology and human papillomavirus testing in cervical cancer screening: joint European cohort study. BMJ.

[8] Wentzensen N, Arbyn M, Berkhof J, Bower M, Canfell K, Einstein M (2017). Eurogin 2016 roadmap: how HPV knowledge is changing screening practice. Int J Cancer.

[9] Iacobellis M, Violante C, Notarachille G, Simone A, Scarfi R, Giuffre G (2018). Clinical validation of REALQUALITY RQ-HPV screen according to the international guidelines for human papillomavirus DNA test requirements for cervical screening. Virol J.

[10] COMMITTEE ON PRACTICE (2016). B-G. practice bulletin no. 168: cervical Cancer screening and prevention [J]. Obstet Gynecol.

[11] Preventive Services Task Force US, Curry SJ, Krist AH, Owens DK, Barry MJ, Caughey AB (2018). Screening for cervical cancer: U.S. Preventive Services Task Force recommendation statement. JAMA.

[12] Huh WK, Ault KA, Chelmow D, Davey DD, Goulart RA, Garcia FA (2015). Use of primary high-risk human papillomavirus testing for cervical cancer screening: interim clinical guidance. Gynecol Oncol.

[13] Lorincz AT, Castle PE, Sherman ME, Scott DR, Glass AG, Wacholder S (2002). Viral load of human papillomavirus and risk of CIN3 or cervical cancer. Lancet.

[14] Khan MJ, Werner CL, Darragh TM, Guido RS, Mathews C, Moscicki AB (2017). American Society for Colposcopy and Cervical Pathology Colposcopy Standards Colposcopy Standards: role of colposcopy, benefits, potential harms, and terminology for colposcopic practice. J Low Genit Tract Dis.

[15] Steenbergen RD, Snijders PJ, Heideman DA, Meijer CJ (2014). Clinical implications of (epi)genetic changes in HPV-induced cervical precancerous lesions. Nat Rev Cancer.

[16] Uijterwaal MH, Polman NJ, Van Kemenade FJ, Van Den Haselkamp S, Witte BI, Rijkaart D (2015). Five-year cervical (pre)cancer risk of women screened by HPV and cytology testing. Cancer Prev Res (Phila).

[17] Sung YE, Ki EY, Lee YS, Hur SY, Lee A, Park JS (2016). Can human papillomavirus (HPV) genotyping classify non-16/18 high-risk HPV infection by riskstratification?. J Gynecol Oncol.

[18] Kjær SK, Frederiksen K, Munk C, Iftner T (2010). Long-term absolute risk of cervical intraepithelial neoplasia grade 3 or worse following human papillomavirus infection: role of persistence. J Natl Cancer Inst.

[19] Gottschling M, Göker M, Stamatakis A, Bininda-Emonds OR, Nindl I, Bravo IG (2011). Quantifying the phylodynamic forces driving papillomavirus evolution. Mol Biol Evol.

[20] Schiffman M, Hyun N, Raine-Bennett TR, Katki H, Fetterman B, Gage JC (2016). A cohort study of cervical screening using partial HPV typing and cytology triage. Int J Cancer.

[21] Cuzick J, Ho L, Terry G, Kleeman M, Giddings M, Austin J (2014). Individual detection of 14 high risk human papilloma virus genotypes by the PapType test for the prediction of high grade cervical lesions. J Clin Virol.

[22] Schiffman M, Burk RD, Boyle S, Raine-Bennett T, Katki HA, Gage JC (2015). A study of genotyping for Management of Human Papillomavirus-Positive, cytology-negative cervical screening results. J Clin Microbiol.

[23] So KA, Kim MJ, Lee KH, Lee IH, Kim MK, Lee YK (2016). The impact of high-risk HPV genotypes other than HPV 16/18 on the natural course of abnormal cervical cytology: a Korean HPV cohort study. Cancer Res Treat.

[24] So KA, Hong JH, Lee JK (2016). Human papillomavirus prevalence and type distribution among 968 women in South Korea. J Cancer Prev.

[25] Nah EH, Cho S, Kim S, Cho HI (2017). Human papillomavirus genotype distribution among 18,815 women in 13 Korean cities and relationship with cervical cytology findings. Ann Lab Med.

[26] Chen HC, Schiffman M, Lin CY, Pan MH, You SL, Chuang LC (2011). Persistence of type-specific human papillomavirus infection and increased long-term risk of cervical cancer. J Natl Cancer Inst.

[27] Jing L, Zhong X, Huang W, Liu Y, Wang M, Miao Z (2014). HPV genotypes and associated cervical cytological abnormalities in women from the Pearl River Delta region of Guangdong province, China: a cross-sectional study. BMC Infect Dis.

[28] Zhang L, Bi Q, Deng H, Xu J, Chen J, Zhang M (2017). Human papillomavirus infections among women with cervical lesions and cervical cancer in eastern China: genotype-specific prevalence and attribution. BMC Infect Dis.

[29] Guan P, Howell-Jones R, Li N, Bruni L, de Sanjosé S, Franceschi S (2012). Human papillomavirus types in 115,789 HPV-positive women: a meta-analysis from cervical infection to cancer. Int J Cancer.

[30] Asato T, Maehama T, Nagai Y, Kanazawa K, Uezato H, Kariya K (2004). A large case-control study of cervical Cancer risk associated with human papillomavirus infection in Japan, by nucleotide sequencing-based genotyping. J Infect Dis.

[31] Tang Y, Zheng L, Yang S, Li B, Su H, Zhang LP (2017). Epidemiology and genotype distribution of human papillomavirus (HPV) in Southwest China: a cross-sectional five years study in non-vaccinated women. Virol J.

[32] Bernard HU, Burk RD, Chen Z, van Doorslaer K, Zur Hausen H, de Villiers EM (2010). Classification of papillomaviruses (PVs) based on 189 PV types and proposal of taxonomic amendments. Virology.

[33] de Sanjose S, Quint WG, Alemany L, Geraets DT, Klaustermeier JE, Lloveras B (2010). Human papillomavirus genotype attribution in invasive cervical cancer: a retrospective cross-sectional worldwide study. Lancet Oncol.

[34] Bruni L, Diaz M, Castellsagué X, Ferrer E, Bosch FX, de Sanjosé S (2010). Cervical human papillomavirus prevalence in 5 continents: meta-analysis of 1 million women with normal cytological findings. J Infect Dis.

[35] Lee SA, Kang D, Seo SS, Jeong JK, Yoo KY, Jeon YT (2003). Multiple HPV infection in cervical cancer screened by HPVDNAChip. Cancer Lett.

[36] Lai CH, Chao A, Chang CJ, Chao FY, Huang HJ, Hsueh S (2008). Host and viral factors in relation to clearance of human papillomavirus infection: a cohort study in Taiwan. Int J Cancer.

[37] Bae J, Seo SS, Park YS, Dong SM, Kang S, Myung SK (2009). Natural history of persistent high-risk human papillomavirus infections in Korean women. Gynecol Oncol.

[38] Muñoz N, Hernandez-Suarez G, Méndez F, Molano M, Posso H, Moreno V, Murillo R, Ronderos M, Meijer C, Muñoz Á (2009). Persistence of HPV infection and risk of high-grade cervical intraepithelial neoplasia in a cohort of Colombian women. British Journal of Cancer.

[39] Mills Anne M., Paquette Cherie, Castle Philip E., Stoler Mark H. (2015). Risk Stratification By p16 Immunostaining of CIN1 Biopsies. The American Journal of Surgical Pathology.

[40] Carcopino X, Bolger N, Henry M, Mancini J, Boubli L, Olive D (2011). Evaluation of type-specific HPV persistence and high-risk HPV viral load quantitation in HPV positive women under 30 with normal cervical cytology. J Med Virol.

[41] Nobbenhuis Mariëlle AE, Helmerhorst Theo JM, van den Brule Adriaan JC, Rozendaal Lawrence, Voorhorst Feja J, Bezemer P Dick, Verheijen René HM, Meijer Chris JLM (2001). Cytological regression and clearance of high-risk human papillomavirus in women with an abnormal cervical smear. The Lancet.

